# MALDI-TOF MS as an innovative tool for detection of *Plasmodium* parasites in *Anopheles* mosquitoes

**DOI:** 10.1186/s12936-016-1657-z

**Published:** 2017-01-03

**Authors:** Maureen Laroche, Lionel Almeras, Emilie Pecchi, Yassina Bechah, Didier Raoult, Angèle Viola, Philippe Parola

**Affiliations:** 1 URMITE, Aix Marseille Université, UM63, CNRS 7278, IRD 198, INSERM 1095, IHU - Méditerranée Infection, 19-21 Boulevard Jean Moulin, 13385 Marseille Cedex 05, France; 2 Unité de Parasitologie et Entomologie, Département des Maladies Infectieuses, Institut de Recherche Biomédicale des Armées, Marseille, France; 3 Aix Marseille Université, CNRS, Centre de Résonance Magnétique Biologique et Médicale, CRMBM, UMR 733927, bd Jean Moulin, 13385 Marseille Cedex 5, France

## Abstract

**Background:**

Malaria is still a major public health issue worldwide, and one of the best approaches to fight the disease remains vector control. The current methods for mosquito identification include morphological methods that are generally time-consuming and require expertise, and molecular methods that require laboratory facilities with relatively expensive running costs. Matrix-Assisted Laser Desorption Ionization Time-Of-Flight Mass Spectrometry (MALDI-TOF MS) technology, routinely used for bacterial identification, has recently emerged in the field of entomology. The aim of the present study was to assess whether MALDI-TOF MS could successfully distinguish *Anopheles stephensi* mosquitoes according to their *Plasmodium* infection status.

**Methods:**

C57BL/6 mice experimentally infected with *Plasmodium berghei* were exposed to *An. stephensi* bites. For the determination of *An. stephensi* infection status, mosquito cephalothoraxes were dissected and submitted to mass spectrometry analyses and DNA amplification for molecular analysis. Spectra were grouped according to mosquitoes’ infection status and spectra quality was validated based on intensity and reproducibility within each group. The in-lab MALDI-TOF MS arthropod reference spectra database, upgraded with representative spectra from both groups (infected/non-infected), was subsequently queried blindly with cephalothorax spectra from specimens of both groups.

**Results:**

The MALDI TOF MS profiles generated from protein extracts prepared from the cephalothorax of *An. stephensi* allowed distinction between infected and uninfected mosquitoes. Correct classification was obtained in blind test analysis for (79/80) 98.75% of all mosquitoes tested. Only one of 80 specimens, an infected mosquito, was misclassified in the blind test analysis.

**Conclusions:**

Matrix-Assisted Laser Desorption Ionization Time-Of-Flight Mass Spectrometry appears to be a promising, rapid and reliable tool for the epidemiological surveillance of *Anopheles* vectors, including their identification and their infection status.

## Background

Malaria is caused by parasites of the *Plasmodium* genus. These pathogens are transmitted to humans during the blood meal of an infected female *Anopheles* mosquito [1]. Five species of *Plasmodium* are known to infect humans [1] and *Plasmodium falciparum* is the most virulent species for humans, causing cerebral malaria and death in the worst cases [1, 2]. A major weapon against malaria remains vector control, which involves monitoring of *Anopheles* mosquito populations and knowledge of their infection status with regards to *Plasmodium* species.

Currently, the identification of mosquitoes is mainly done by morphological or molecular methods [3]. Morphological identification is a reliable method and yet may be be limited by the requirement of identification keys, specific documentation and entomological expertise. Using these methods can be lengthy if a large number of samples must be identified. Moreover, morphological tools cannot differentiate mosquitoes belonging to a species complex, such as the Gambiae complex, which includes 8 species that are not all *Plasmodium* vectors [6]. Molecular techniques are an alternative. However, they require specific laboratory facilities. They may also be time-consuming and relatively expensive, especially when sequencing is required [4]. The infection status of arthropods can be determined in diverse ways, but three major methods are used for detection of *Plasmodium* in mosquitoes. The microscopic approach, which is routinely used in malaria-endemic countries, entails the observation of live or stained parasites in dissected or crushed mosquitoes [5]. Although it is an affordable and relevant method, it is time-consuming, and conclusions can be operator-dependent [6]. The second approach relies on immunological methods, such as the enzyme-linked immunosorbent assay (ELISA) or direct immunohistochemistry, both methods targeting *Plasmodium* antigens such as the circumsporozoite protein (CSP). Antibodies are frequently used to distinguish *Plasmodium*-infected from parasite-free *Anopheles.* However, this method presents several limitations, such as the diversity of antibodies required for the specific detection of *Plasmodium* species, the risk of cross-reaction with close parasite species by antibodies, or the difficulty of data interpretation when the signal-to-noise ratio is low [7]. Molecular methods such as standard PCR, nested-PCR and qPCR are adequate and sensitive for *Plasmodium* detection on whole and pooled mosquitoes [10, 11], but the preparation of the samples and cost of the reagents may limit their use. Thus, the development of a consensual alternative tool for rapid and inexpensive identification of mosquitoes, and also for the determination of their infection status, appears important for the development of malaria epidemiological surveillance.

In recent years, matrix-assisted laser desorption/ionization time-of-flight mass spectrometry (MALDI-TOF–MS) has been used for the identification and classification of microorganisms [15, 16], and it has also been applied in the identification of arthropods [7], including mosquitoes [17–19]. It requires the creation of a reference spectra database obtained from organisms unambiguously identified by morphology and molecular biology reference methods. More recently, MALDI-TOF has been described as a promising alternative for the detection of microorganisms in arthropods. Indeed, it was reported that this innovative tool could differentiate rickettsiae-infected and non-infected ticks, using only tick legs [8] or tick haemolymph [9]. The dual identification of arthropod species and infection status at the same time by MALDI-TOF could be revolutionary for vector monitoring and entomological diagnosis.

Based on these promising results, the goal of the present study was to assess whether MALDI-TOF could be used to distinguish *Plasmodium*-infected *Anopheles* from uninfected mosquitoes. To do so, mosquitoes were infected by feeding on C57BL/6 mice experimentally infected with *Plasmodium berghei* (ANKA strain). MS spectra from molecularly validated infected and non-infected mosquitoes were then compared to evaluate the ability of mass spectrometry to distinguish the two categories.

## Methods

### Mice and ethics statement

Female C57BL/6 J mice from the Charles River Laboratories (Saint-Germain-Nuelles, France) (8–10 week old, 20–25 g body weight) were maintained at 23–25 °C with a 12 h light/12 h dark cycle, with free access to food and water. Animal studies were conducted in agreement with the directive 2010/63/EU of the European Parliament and of the Council of 22 September 2010 and in agreement with decree No. 2013-118 of 1 February 2013 from the French Government. The study was approved by the local Ethics Committee (Comité d’Ethique en Expérimentation Animale Marseille 14) of Marseille, France.

### Murine model of experimental malaria with *Plasmodium berghei*

Six female mice were infected with the same batch of *P. berghei* (ANKA strain) parasites by intraperitoneal injection of 2 × 10^6^ parasitized red blood cells per mouse, after validation of the cell titer on KOVA-slides (Fisher Scientific, Illkirch, France). Parasitaemia, expressed as the percentage of parasitized red blood cells, was determined daily from day 3 post-infection by Giemsa-stained tail-blood smears, as previously described [10]. Gametocytaemia, expressed as the percentage of parasites in gametocyte form, was determined as well. Animals were monitored daily for clinical signs of cerebral malaria until mouse sacrifice. The main focus was on weight loss, which was the main clinical manifestation detectable during animal follow-up. Four mice of the same age and same breeding conditions were used as controls.

### Anopheles rearing

Only *Anopheles stephensi* mosquitoes were used in this study, and all were from the Institut Pasteur laboratory rearing facility. They were reared in the laboratory (Aix-Marseille University, Marseille, France) using standard methods, with temperature of 26 ± 1 °C, relative humidity of 80 ± 10% and a 12 h light/12 h dark cycle in incubators (Panasonic cooled incubator). Larvae were reared until the pupal stage in trays containing 1 litre of distilled water supplemented with fish food (TetraMin Baby, Tetra GmbH, Herrenteich, Germany). Pupae were collected daily and transferred to a mosquito cage (Bug Dorm 1, BioQuip products). Adults were fed with a 10% glucose solution. For egg production, blood meals were given through a Parafilm-membrane and an artificial feeding device (Hemotek membrane feeding systems, Discovery Workshops UK), using fresh human blood [11]. Female *An. stephensi* planned to be used for this work were isolated and fed only with a 10% sugar solution from their emergence until their mouse blood meal 4 days later. After the blood meal on mice, Plasmodium exposed mosquitoes were reared at temperature of 21 ± 1 °C, relative humidity of 80 ± 10% and a 12 h light/12 h dark cycle in incubators.

### Anopheles feeding and sampling

Based on parasitaemia greater than 1% and the appearance of gametocytes in blood, mice were anesthetized by intraperitoneal injection of ketamine (90 mg/kg) and xylazine (10 mg/kg). Immediately after complete anesthesia, mice abdomens were thoroughly shaved and disinfected with ethanol. Mice were then placed directly onto mosquito cages to allow engorgement for 1 h. Mosquitoes were killed 18 days after their infective blood meal by storage at −20 °C until further utilization.

### Sample preparation for MALDI-TOF MS

Mosquitoes’ cephalothoraxes (head and thorax) were selected for MALDI-TOF MS. Eighteen days post mouse-blood feeding, each mosquito was briefly washed in 70% ethanol, rinsed in sterile water, dried and dissected. Glass powder and 50 µl of HPLC-grade water were added to mosquito cephalothoraxes (CTs) before crushing them using the TissueLyser instrument (Qiagen, Hilden, Germany) at a frequency of 30 movements per second for 60 s. This cycle was repeated twice and followed by a single 3 min cycle. The crushed CTs were centrifuged at 10,000 rpm for 30 s and 1 µL of the supernatant of each sample was carefully dropped on a MALDI-TOF target plate in quadruplicate (Bruker Daltonics, Wissembourg, France) [12]. Each spot was then recovered with 1 µL CHCA matrix solution composed of saturated α-cyano-4-hydroxycynnamic acid (CHCA) (Sigma, Lyon, France), 50% acetonitrile (v/v), 2.5% trifluoroacetic acid (v/v) (Aldrich, Dorset, UK) and HPLC-grade water. After drying for several minutes at room temperature, the target plate was introduced into the Microflex LT MALDI-TOF Mass Spectrometer device (Bruker Daltonics, Germany) for analysis. To control loading on mass spectra steel, matrix quality and MALDI-TOF apparatus performance, matrix solution only was also loaded in duplicate onto each MALDI-TOF plate with or without a *E. coli* bacterial test standard (Bruker protein Calibration Standard I).

### DNA extraction and molecular analysis

Infection by *P. berghei* was simultaneously assessed in all the samples using MALDI-TOF and molecular biology. To do so, the remaining supernatant and debris of crushed CTs after MS plate loading were used for molecular determination of the infection status of the collected mosquitoes. Crushed CTs were vortexed with 200 µL of G2 buffer solution containing 20 µL of proteinase K (15 mg/mL) (Qiagen, Hilden, Germany) and incubated at 56 °C overnight prior to DNA extraction. DNA extractions from individual mosquito samples were performed with the Qiagen EZ1 Advanced XL Robot with respective Qiagen kits (Qiagen, Hilden, Germany), according to the manufacturer’s recommendations. DNA from each sample was eluted in 50 µL of Tris EDTA (TE) buffer (Qiagen, Hilden, Germany) and either immediately used or stored at −20 °C. Primers and probe targeting a 189 bp region of the 18S gene of all *Plasmodium* species were used to detect *P. berghei* DNA by real-time quantitative PCR (Table 1). Real-time quantitative PCR was carried out according to the manufacturer’s protocol using a CFX Connect™ Real-Time PCR Detection System (Bio-Rad) with the Eurogentec Takyon qPCR kit. DNA extracted from *P. berghei*-infected mouse blood was used as positive control. DNA extracted from mosquitoes of the non-infected group and DNA-free PCR mix were used as PCR negative controls. Standard PCR targeting a 710 bp region of the invertebrate cytochrome oxidase I (*COI*) gene [13] was performed on a randomly selected batch of 21 exposed but PCR negative mosquitoes.

**Table 1:** Sequences of primers and probe used for the quantitative PCR detection of *P. berghei* in mosquitoes

Target	Primer name	Sequence
*18S* gene All *Plasmodium* species	18S_F	5′-AGGCAACAACAGGTCTGTGA-3′
	18S_R	5′-GCAATAATCTATCCCCATCACG-3′
	18S_P	5′-6FAM- GAACTAGGCTGCACGCGTGCTACA-3′

Primers and probe targeting a 189 bp sequence of all *Plasmodium* species 18S gene used for the detection of *Plasmodium berghei* ANKA in mosquitoes

### MALDI-TOF–MS parameters

Protein mass profiles were obtained using a Microflex LT MALDI-TOF Mass Spectrometer (Bruker Daltonics, Germany), with detection in the linear positive-ion mode at a laser frequency of 50 Hz within a mass range of 2–20 kDa. The acceleration voltage was 20 kV, and the extraction delay time was 200 ns. Each spectrum corresponds to ions obtained from 240 laser shots performed in six regions of the same spot and automatically acquired using the AutoXecute of the Flex Control v.2.4 software (Bruker Daltonics). The average spectrum profiles obtained were visualized with Flex analysis v.3.3 software and exported to ClinProTools software v.2.2 and MALDI-Biotyper v.3.0. (Bruker Daltonics, Germany) for data processing (smoothing, baseline subtraction, and peak picking) as previously described [14].

### MALDI-TOF analysis and database creation

Spectrum quality was validated by assessing its general intensity, the smoothness of the peaks, the flatness of baseline and its reproducibility compared to other spectra of the same categories. Poor quality spectra were excluded from the analysis. Spectra reproducibility of CTs from *An. stephensi* specimens molecularly defined as *P. berghei*-infected or non-infected was evaluated by comparing the average spectra of each sample within its respective group using ClinProTools 2.2 and Flex analysis v.3.3 software (Bruker Daltonics, Germany). Reference spectra (MSP, Main Spectrum Profile) were generated by the automated function of the MALDI-Biotyper software v3.0 (Bruker Daltonics, Germany) by combining the results of the spectra of at least two specimens per condition in order to create a database. MSP were produced based on an unbiased algorithm, taking into consideration the peak position, intensity and frequency. Spectra from thirty control mosquitoes were selected based on their reproducibility and intensity for all further MS analyses. Random spectra of good intensity and good reproducibility of specimens from each batch (infected/non-infected) were loaded in MALDI-Biotyper 3.0 software to create a reference spectra database. Twelve spectra from control *An. stephensi* were added to the custom database, as well as six spectra of infected specimens and two spectra of exposed but PCR-negative specimens.

### MALDI-TOF MS biomarker mass set

To determine the CT differential peaks between *An. stephensi*, infected or not with *P. berghei*, the MS spectra from both groups were loaded into ClinProTools 2.2 software. The software was used to generate a peak list for each group in the 2–20 kDa mass range and to identify discriminating peaks. The parameter settings in ClinProTools 2.2 software for spectra preparation were as follows: a resolution of 800 ppm; a noise threshold of 3.00 (intensity arbitrary unit); a maximum peak shift of 800 ppm and a match to calibration peaks of 20%. For the peak calculation, peak peaking was performed on single spectra with a signal-to-noise threshold of 3.00 and an aggregation of 800 ppm. The spectra were then analysed with the genetic algorithm (GA) model, which displayed a list of discriminating peaks [6]. Manual inspection and validation of the selected peaks by the operator gave a “recognition capability” (RC) value together with the highest “cross-validation” (CV) value. These values reflect the ability to distinguish the different spectra groups based on the chosen discriminatory peaks. Genetic Algorithm settings are optimized to provide the highest RC and CV values. All reference spectra from infected and non-infected *An. stephensi* were imported into ClinProTools software, in order to detect the presence of discriminating peaks between the spectra of infected and non-infected CTs.

### Blind tests

The remaining CT spectra of each group after exclusion of those entered in the database were submitted to a blind test analysis. This blind test was performed against our in-lab database, composed of the present CT spectra from *P. berghei*-infected and non-infected *An. stephensi*, and also spectra from other arthropod species listed in Table 2 [12, 15–18]. The identification level of significance was determined using the log score values (LSVs) given by the MALDI-Biotyper software v.3.3., corresponding to a matched degree of signal intensities of mass spectra of the query and the reference spectra. LSVs ranged from 0 to 3. LSVs allow good evaluation of the reproducibility between a queried spectrum and a reference spectrum as it is the result of thorough comparison of peaks position and intensity between those two spectra. A LSV was obtained for each spectrum of the samples tested blindly.

**Table 2:** List of the arthropod species present in our in-lab MALDI-TOF MS database

Mosquitoes	*Anopheles gambiae, A. coluzzi, An. funestus, An. ziemanni, An. arabiensis, An. wellcomei, An. rufipes, An. pharoensis, An. coustani, An. claviger, An. hyrcanus, An. maculipennis, Culex quinquefasciatus, Cx. pipiens, Cx. modestus, Cx. insignis, Cx. neavei, Aedes albopictus, Ae. excrucians, Ae. vexans, Ae. rusticus, Ae. dufouri, Ae. cinereus, Ae. fowleri, Ae. aegypti, Ae. caspius, Mansonia uniformis, Orthopodomyia reunionensis, Coquillettidia richiardii and Lutzia tigripes*
Ticks	*Amblyomma. variegatum infected or not by Rickettsia africae, Rh. sanguineus, Rh. bursa, Rh. sulcatus, Hyalomma m. rufipes, I. ricinus, I. hexagonus, D. marginatus infected or not by R. slovaca, D. reticulates, Haemaphysalis concinna*
	In ethanol: *Am. cohaerens, Am. gemma, Am. variegatum, Ha. leachi, Hy. m.rufipes. Hy. truncatum, Hy. detritum, Rh. decoloratus, Rh. bergeoni, Rh e. evertsi, Rh. praetextatus, Rh. pulchellus, Rh. sanguineus, Rh. microplus, Rh. annulatus, Rh. turanicus, Rh.bursa*
Triatomines	*Triatoma infestans, Rhodnius prolixus, Rh. pictipes, Rh. robustus, Eratyrus mucronatus, Panstrongylus geniculatus*
Bedbugs	*Cimex lectularius*
Fleas	*Ctenocephalides felis, Ct. canis, Archaeopsylla erinacei, Xenopsylla cheopis* and *Stenoponia tripectinata*
Mites	*Leptotrombidium chiangraiensis, L. imphalum, L. deliense*
Sandflies	*Phlebotomus papatasi, P. (Larrousius) longicuspis), P. (Larrousius) perfiliewi, P. (Larrousius) perniciosus, P. (Paraphlebotomus) sergenti, Sergentomyia minuta*
Lice	*Damalinia bovis, D. caprae, D. ovis, Haematopinus eurysternus, Linognatus vituli, L. africanus, Pediculus humanus*

Mosquito, tick, triatomine, bedbug reference spectra were obtained from legs protein extracts. Flea reference spectra were obtained from the whole body without abdomen protein extracts. Sandfly reference spectra were obtained from thorax, wings and legs protein extracts. Louse reference spectra were obtained from half of the body protein extracts. These species include field specimens or from insectary breeding, but also specimens collected from patients

## Results

### Assessment of *Plasmodium berghei* mouse infection and Anopheles feeding

**Fig. 1:**
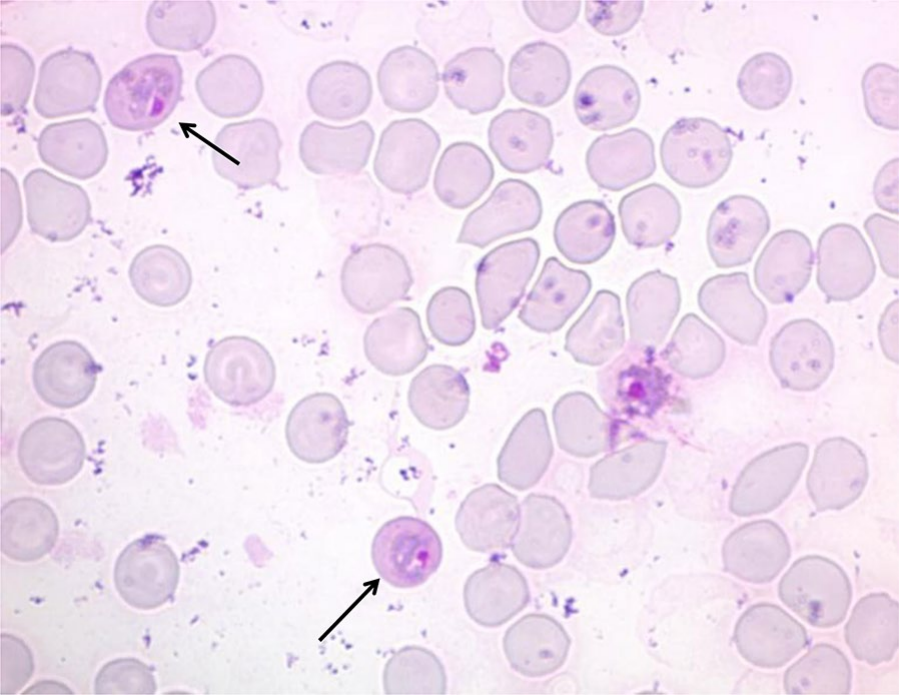
Giemsa-stained mouse tail-blood smear performed 4 days post-infection showing gametocytes. Gametocyte-infected cells are indicated by *black arrows*

All six mice were successfully infected with *P. berghei*-parasitized red blood cells as demonstrated by parasitaemia on follow-up and loss of body weight. While the blood smears performed at day 3 post-infection did not reveal any parasitized red blood cells, parasitaemia was detected in all mice and estimated at approximately 1.5% (mean + SD:1.5 ± 0.35) on day 4 after *P. berghei* inoculation (90 h post-infection). Approximately 5% (mean + SD: 5.08 ± 1.4) of the parasites detected were in gametocyte form (Fig. 1). This time point was selected for immediate mosquito engorgement, because a rapid degradation of gametocytes was noticed.

One hundred and seventy female *Anopheles* mosquitoes were then allowed to engorge on the *Plasmodium*-infected anesthetized mice, while 145 mosquitoes fed on 4 healthy mice. After 1 h of feeding, 156 mosquitoes were engorged from the infected mice and 111 were engorged from parasite-free mice.

### Molecular analysis

Eighteen days after the infective blood meal, 50 control *Anopheles* (fed on healthy mice) and 60 *Plasmodium*-exposed mosquitoes were collected. DNAs were extracted from the crushed CTs, and 20 of 60 parasite-exposed mosquito DNAs were positive in real-time PCR targeting of a region of the *18S* gene of the *Plasmodium* species with cycle threshold (Ct) values ranging from 24.99 to 34.68. All control mosquitoes tested were negative for *Plasmodium* detection.

All mosquitoes tested by *COI*-standard PCR for validation of DNA extraction were positive.

### MALDI-TOF analysis

**Fig. 2:**
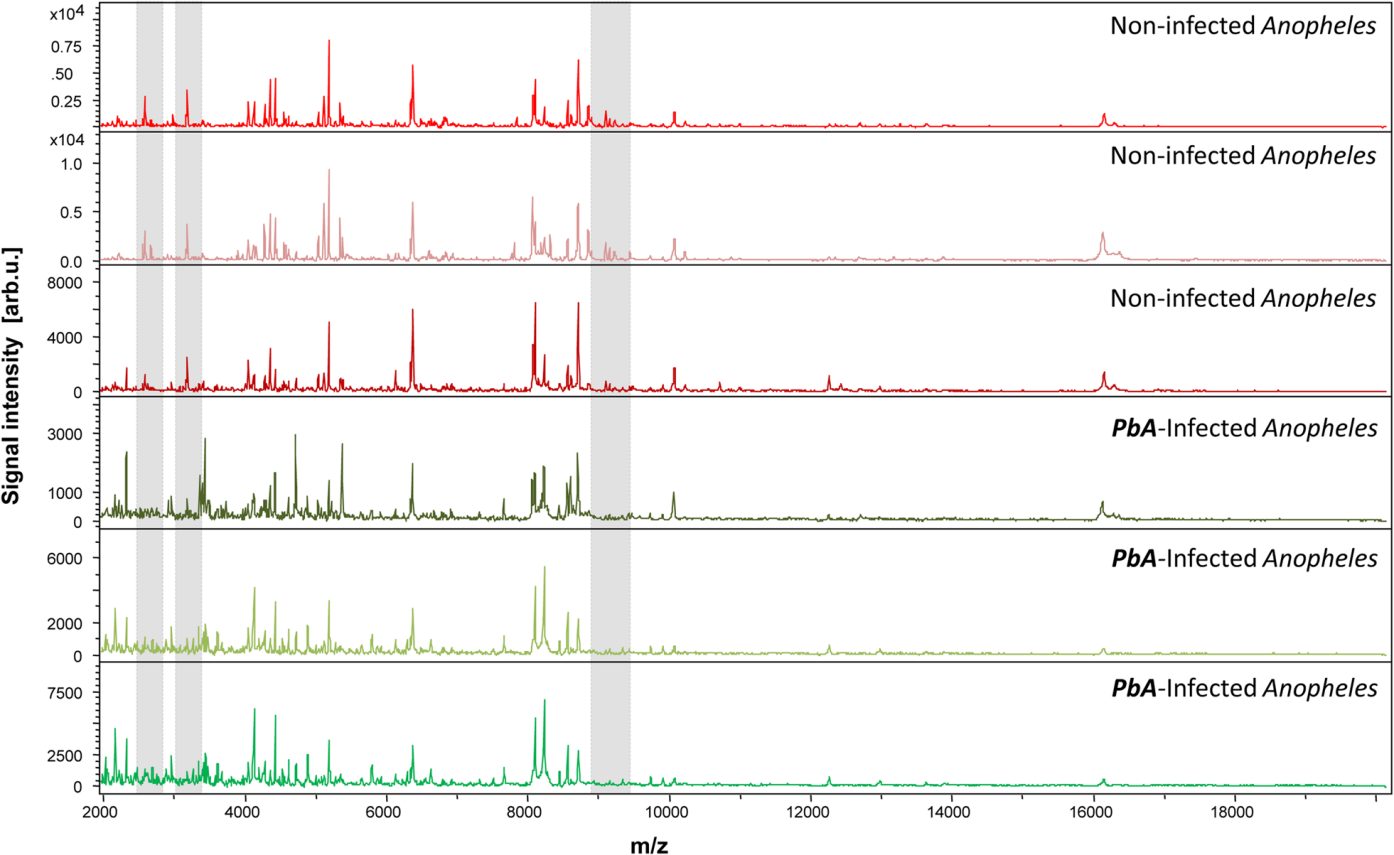
Representative MS spectra of non-infected and *Plasmodium berghei*-infected *Anopheles stephensi* cephalothoraxes using Flex analysis 3.3 software. a.u., arbitrary units; m/z, mass-to-charge ratio. *Vertical bar* highlights of distinct spectral regions between infected and uninfected mosquitoes

To appraise water-crushing reproducibility, CT MS spectra of non-infected *An. stephensi* were submitted to MALDI-TOF MS analysis. Afterwards, the comparison of the MS spectra of infected and non-infected *An. stephensi* with Flex Analysis and ClinProTools software indicated correct reproducibility of the protein profiles of CTs from mosquitoes of the same group (Fig. 2). Nevertheless, mild variations were still observed between spectra of the same group. For this reason, spectra selection for the database was supported by the creation of a dendrogram. Dendrograms are based on the results of Composite Correlation Index matrix (CCI). CCIs are calculated by dividing spectra into intervals and comparing these intervals across a data set. The composition of correlations of all intervals provides the CCI, which is used a parameter that defines the distance between spectra. A CCI match value of 1 represents complete correlation, whereas a CCI match value of 0 represents an absence of correlation. Spectra chosen for the database upgrading were from different clusters, therefore representing the whole group, despite its relative heterogeneity.

**Fig. 3:**
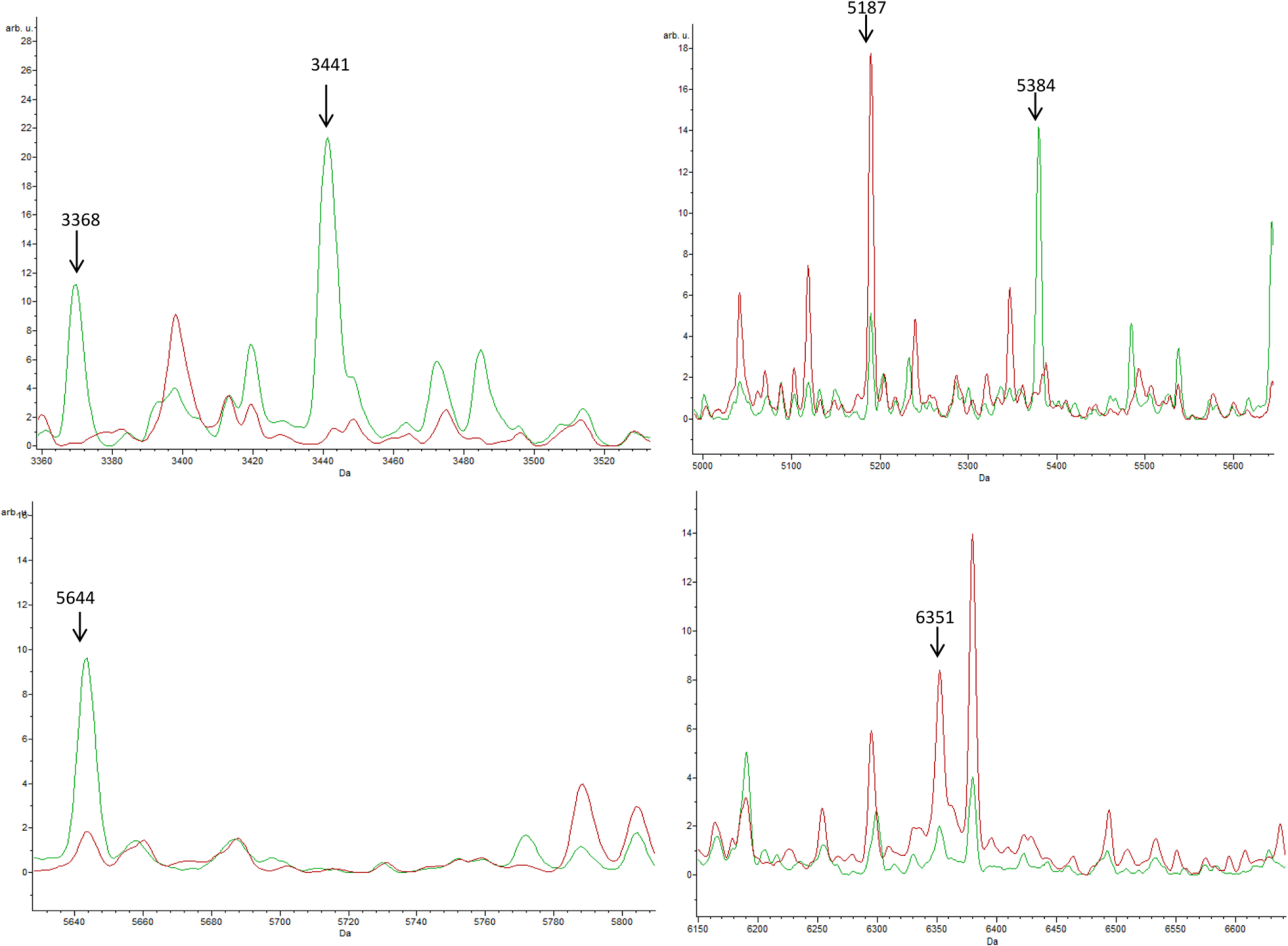
Comparison of MALDI-TOF MS profiles of mosquitoes infected or not by *Plasmodium berghei* using ClinProTools software. *Red* and *green* profiles correspond to average spectrum of control and infected mosquitoes respectively. *Peak masses* (Da) are indicated with an* arrow* above the referred peak. arb.u., arbitrary units; *m/z*, mass-to-charge ratio

Visual comparison of MS profiles between the two groups revealed a few specific distinct peaks. Precise determination of discriminatory peaks between the two categories was then assessed using the ClinProTools software. In order to distinguish mosquitoes according to their infection status, 20 spectra from *P. berghei*-infected mosquitoes were compared to 30 spectra from non-infected mosquitoes using the Genetic Algorithm (GA) tool. After verification of the peak report in the average spectrum, 13 discriminatory masses were determined (Table 3; Fig. 3). The selected peaks of the Genetic Algorithm displayed recognition capability and cross validation values of 100 and 98.72%, respectively.

**Table 3:** Comparison of MALDI-TOF profiles of uninfected and *P. berghei*-infected *An. stephensi* mosquitoes and determination of distinguishing peak masses

Mass *m/z* (Da)	*An. stephensi*	*An. stephensi* infected with *Plasmodium berghei* ANKA
3368	No	Yes
3441	No	Yes
4430	Yes	No
5084	Yes	No
5187	Yes	No
5384	No	Yes
5644	No	Yes
5803	No	Yes
6351	Yes	No
6790	Yes	No
7521	No	Yes
8448	Yes	No
12,264	No	Yes

Determination of the CT differential peaks between *An. stephensi* infected or not with *P. berghei* obtained with the Genetic Algorithm tool of ClinProTools 2.2 software. All mosquitoes are from the same laboratory rearing

The upgraded MS reference database containing the 20 new spectra added in this study was queried blindly, resulting in 100% correct species-level identification and 98.75% correct identification regarding the infection status for all tested samples, with LSVs ranging from 1.347 to 2.908. A total of 13/14 specimens considered as infected based on molecular analysis matched with reference spectra of *P. berghei*-infected *An. stephensi*. All control specimens were identified as non-infected by MALDI TOF MS. Also, mosquitoes exposed to parasitized mice but *Plasmodium* PCR-negative were identified as non-infected by MALDI TOF MS. Indeed, 100% of these spectra matched with the reference spectra of non-infected *An. stephensi* (Table 4).

**Table 4:** Comparison of molecular biology and mass spectrometry performances for the detection of *Plasmodium berghei* in *An. stephensi* mosquitoes

	Controls	PCR+	PCR-	TOTAL
Total number of spectra	42	20	38	100
Number of spectra introduced in DB	12	6	2	20
Number of spectra tested blinded against DB	30	14	36	80
Percentage of MALDI-TOF consistency with PCR	30/30 (100%)	13/14 (92.8%)	36/36 (100%)	79/80 (98.75%)

Results of MALDI-TOF blinded tests were based on the highest log score values and obtained with the MALDI BioTyper software. Controls: *Anopheles* fed on non-infected mice, PCR + : mosquitoes fed on infected mice and for which *P. berghei*-PCR was positive, PCR − : mosquitoes fed on infected mice and for which *P. berghei*-PCR was negative. *DB* database

## Discussion

The present work shows preliminary evidence that MALDI-TOF technology can be used for *Plasmodium* detection. *Anopheles stephensi* and *Plasmodium berghei* ANKA is one of the most frequently used pairs for experimental models involving this murine parasite [26].

The blood smears performed on day 4 post-infection showed a low parasitaemia, as the average of the six blood smears was approximately 1% of infected red blood cells. However, approximately 5% of the parasites were in gametocyte form, some of them starting to deteriorate, highlighting the very short time window during which mosquitoes could be fed on the mice. Published literature suggests that peak gametocytaemias for *P. berghei* ANKA takes place around day 4 post-infection [27], with possible variations depending on the mouse strain and environmental parameters. However, the gametocytaemias described were always much lower than those observed in this work [19].

*Plasmodium* detection was performed on samples collected 18 days after the infective blood meal. The *18S* gene-qPCR positivity cut-off was chosen according to the Ct value of the positive control, which was formerly validated by microscopy. The blood used as positive control was previously checked by microscopy for the presence of parasites in fresh blood and Giemsa-stained blood smears. As this cut-off was established at 35.6 Ct value empirically, positivity of samples with Ct values slightly superior to 35 could not be excluded with certainty. However, for the proof of concept of this work, we decided to use mosquitoes for which the infection status was certain. Mosquito sampling was done 18 days after the infective blood meal to ensure that the parasite had already undergone several multiplications and had completed its migration towards the salivary glands [29].

The in-lab MALDI TOF mosquito database was initially built using mosquito legs crushed manually with pestles [12]. However, for determination of infection status in this study, analyses were performed on CTs, where parasites are located when the mosquito is infected [5]. Moreover, standardization of the protocol required elimination of the manual sample preparation method and development of an automated crushing technique. The TissueLyser crushing device is traditionally used for mechanical cell disruption [20]. It was recently adapted for the standardization of MALDI-TOF preparation of arthropod samples, including *An*. *gambiae* and *An. stephensi* CTs (unpublished). This automated crushing technique provides reproducible MS spectra for *An. stephensi* CTs.

Molecular biology using a whole mosquito is a standard and validated technique for the detection of *Plasmodium* parasites [21]. Mosquitoes were considered as infected when parasites were detected in the CT by qPCR with Ct values inferior to 35, and DNA extractions were thoroughly validated by detection of the mosquito *COI* gene by standard PCR. Because PCR and mass spectrometry had to be performed on the same mosquito compartment, protein extraction was performed in high performance liquid chromatography-grade water instead of the standard solution of formic acid and acetonitrile, responsible for cell wall disruption and protein extraction, respectively [22]. Sample crushing in water already showed MALDI-TOF results comparable to crushing in formic acid in previous entomological studies [23]. However, the absence of acid impairs protein extraction and could explain the mild spectra variability that was observed within the same group, but also the poor quality of the spectra that were excluded from this study. Comparison of spectra from CTs crushed in water with acid/acetonitrile-derived spectra revealed high similarity of the profiles, with much higher quality and reproducibility for the samples crushed in acid solution. (unpublished). Here, spectra used to upgrade the database were thoroughly selected to be representative of the whole group, despite its variability. The single misidentification observed in this study could be attributable to the water crushing and its resulting variability. Comparison between the average profiles of the infected mosquitoes and non-infected mosquitoes revealed several peaks distinguishing the two categories. These modifications in the mosquito’s global proteome could be attributable to additional proteins linked to the parasite presence but also to the mosquito’s immune response to infection. Indeed, while it was admitted for a long time that infections are neutral for the arthropod vector, it has been very well described that Plasmodium infection triggers a significant immune response in Anopheles mosquitoes. During this response, several genes coding for proteins involved in innate immunity are upregulated, but some of them are also downregulated, which could explain why some peaks are disappearing on the average profile of infected mosquitoes [24, 25].

Besides the two main limitations of MALDI-TOF technology, which are the cost of the device and the comprehensiveness of the database, this high-throughput technique allows analysis of a large number of samples at limited cost compared to current methods.

This technique is already established in Africa, since bacteriology laboratories in Dakar, Senegal are now equipped with MALDI-TOF devices for clinical microbiology purposes. Productivity has consequently been increased there, and it has contributed to an intense development of these laboratories in the field of microbiology. With a running cost lower than $1 per sample, this technology remains less expensive than traditional methods, despite the high initial cost of the devices [26, 27]. Also, as previously mentioned by Yssouf et al. [23], the spectra files are available on request and transferable to any Bruker MALDI-TOF device, thus excluding any additional cost associated with the construction of this database (e.g., molecular biology). Since the application of MS to entomological studies does not lead to additional cost, the protocol described here remains entirely affordable. In fact, entomological applications have already been developed in Dakar, since MS identification of *Culicoides* was finalized there [28]. This preliminary work remains to be validated with field samples. Adjustments and upgrading of our database will be necessary in order to include other reference spectra, such as *Anopheles gambiae* infected or not by *P. falciparum*. It would be interesting to assess the consistency of MS spectra from diverse mosquito populations, and also the performance of the identification associated with plasmodial detection for species belonging to the same complex. The detection of the same plasmodial infection in different mosquito species was not evaluated here, nor the detection of different *Plasmodium* species in the same *Anopheles* species. It would be interesting to do so in further studies, along with an assessment of the ability of MALDI-TOF to identify *Plasmodium* mixed infections. If proved relevant, this technique will be a rapid and convenient tool for the monitoring of the mosquito population in these *Plasmodium*-endemic areas, which is crucial for determination of at-risk populations and for subsequent care. This tool will allow analysis of large numbers of mosquito specimens at limited cost, quickly providing all the necessary information, such as mosquito identification and *Plasmodium* infection status.

## Conclusion

Matrix-Assisted Laser Desorption Ionization Time-Of-Flight appears to be a promising tool for the detection of *Plasmodium* spp. in mosquitoes, in addition to its ability to identify mosquitoes at the species level. This approach does not require knowledge of entomology or molecular biology once the database is comprehensive. However, any upgrade of the database will always require precise identification, initially provided by morphology and molecular biology. This high-throughput, fast and low-cost method makes it an appropriate technique for epidemiological studies and monitoring of *Anopheles* populations in *Plasmodium*-endemic areas, with the concomitant identification of mosquito species and their pathogens. The rapid identification of at-risk populations can provide a valuable advantage for vector control, which may include targeted installations of collective and individual protection against mosquitoes.
